# Expression of Transforming Growth Factor Beta Isoforms in Canine Endometrium with Cystic Endometrial Hyperplasia–Pyometra Complex

**DOI:** 10.3390/ani11061844

**Published:** 2021-06-21

**Authors:** Marta Rybska, Magdalena Woźna-Wysocka, Barbara Wąsowska, Marek Skrzypski, Magdalena Kubiak, Beata Błaszak, Anna Łukomska, Tomasz Nowak, Jędrzej M. Jaśkowski

**Affiliations:** 1 Department of Preclinical Sciences and Infectious Diseases, Poznan University of Life Science, Wołyńska 35, 60-637 Poznan, Poland; barbara.wasowska@up.poznan.pl (B.W.); anna.lukomska@up.poznan.pl (A.Ł.); 2 Department of Medical Biotechnology, Institute of Bioorganic Chemistry Polish Academy of Sciences, Noskowskiego 12/14, 61-704 Poznan, Poland; magda.wozna@wp.pl; 3 Department of Local Physiological Regulations, Institute of Animal Reproduction and Food Research of Polish Academy of Sciences, Tuwima 10, 10-747 Olsztyn, Poland; 4 Department of Animal Physiology, Biochemistry and Biostructure, Poznan University of Life Sciences, Wołyńska 35, 60-637 Poznan, Poland; marek.skrzypski@up.poznan.pl; 5 Department of Internal Diseases and Diagnostics, Poznan University of Life Sciences, Wołyńska 35, 60-637 Poznan, Poland; magdalena.kubiak@up.poznan.pl; 6 Department of Tumour Pathology, Greater Poland Cancer Centre, Garbary 15, 61-866 Poznan, Poland; b.a.blaszak@gmail.com; 7 Department of Genetics and Animal Breeding, Poznan University of Life Sciences, Wołyńska 35, 60-637 Poznan, Poland; tomasz.nowak@up.poznan.pl; 8 Department of Diagnostics and Clinical Sciences, Institute of Veterinary Medicine, Nicolaus Copernicus University, Gagarina 7, 87-100 Torun, Poland; jmjaskowski@umk.pl

**Keywords:** pyometra, cystic endometrial hyperplasia, canine, endometrium, uterus, transforming growth factor beta

## Abstract

**Simple Summary:**

Pathomorphological changes and functional disorders of the uterus have long been a significant problem in the reproduction of dogs. The most commonly identified uterine disorders leading to permanent loss of fertility in dogs include cystic endometrial hyperplasia (CEH) and pyometra. These diseases may occur jointly as a CEH–pyometra complex. Despite numerous studies, the etiology of this disease remains unclear. TGF-β is considered to be one of the key factors in pathophysiological uterine disorders. The results indicate the significant expression of TGF-β1 in endometrial tissues in bitches affected by CEH–pyometra complex. Consequently, among all TGF-β isoforms, TGF-β1 is a potential biomarker involved in the regulation of a dog’s endometrium with proliferative and degenerative changes.

**Abstract:**

Cystic endometrial hyperplasia (CEH) and pyometra are the most frequently diagnosed uterine diseases affecting bitches of different ages. Transforming growth factor beta (TGF-β) has been classified in females as a potential regulator of many endometrial changes during the estrous cycle or may be involved in pathological disorders. The aim of this study was to determine the expression of TGF-β1, -β2 and -β3 in the endometrium of bitches suffering from CEH or a CEH–pyometra complex compared to clinically healthy females (control group; CG). A significantly increased level of TGF-β1 mRNA expression was observed in the endometrium with CEH–pyometra compared to CEH and CG. Protein production of TGF-β1 was identified only in the endometrium of bitches with CEH–pyometra. An increase in TGF-β3 mRNA expression was observed in all the studied groups compared to CG. The expression of TGF-β2 mRNA was significantly higher in CEH and lower in CEH–pyometra uteri. The results indicate the presence of TGF-β cytokines in canine endometrial tissues affected by proliferative and degenerative changes. However, among all TGF-β isoforms, TGF-β1 could potentially be a key factor involved in the regulation of the endometrium in bitches with CEH–pyometra complex.

## 1. Introduction

The most frequently diagnosed uterine disease complex affecting bitches of all ages is cystic endometrial hyperplasia (CEH) or pyometra, diseases which together can create “CEH–pyometra complex”[1]. These diseases are the most common reproductive disorders in older bitches, although have also been reported in middle-age diestrus females [1,2,3]. The etiology of the processes underlying this reproductive tract pathology is multifaceted and not yet fully explained.

According to various studies, an important element allowing not only for detailed diagnosis but also for the determination of the mechanisms of disease development is the study of the gene expression profile of selected factors in uterine tissue. Most of the genes activated in pyometra are associated with chemokines, cytokines, extravasation of inflammatory cells, antibacterial activity, complement systems and innate immune responses [4,5,6,7].

Multifunctional polypeptides that have various effects on cells include the transforming growth factor beta (TGF-β) family. Involved in the regulation of gene expression, these molecules play an important role in the processes of cell growth and differentiation [8]. All three TGF-β isoforms are encoded by different genes. These genes show 80% similarity with sequences and are located on different chromosomes, both in dogs and humans [9]. TGF-β1, -β2 and -β3 are composed of two subunits containing 112 amino acids, bound by disulfide bonds. In mammals, these proteins are characterized by an almost 70% identical sequence of amino acids of similar molecular weight: TGF-β1 (15 kDa), TGF-β2 (12.5 kDa) and TGF-β3 (12 kDa) [9,10]. In vitro studies have shown that these isoforms connect and create similar signal pathways by activating the same TGF-β receptors. Consequently, they induce similar effects in terms of cellular interaction. The high degree of conservativeness of the described isoforms in many species during evolutionary processes indicates their important regulatory functions [11,12].

Signaling with the participation of factors belonging to the TGF-β family is necessary to maintain proper reproductive functions, and deregulation of this process may have negative effects that lead to many reproductive diseases and cancers [13]. Due to their cellular interaction and the molecular processes associated with their proliferation, differentiation, angiogenesis and immunomodulation, TGF-β polypeptides have been classified as potential regulators of many changes occurring in the ovaries and endometrium [14,15,16]. According to current knowledge, ovarian activity is also important for the development of the endometrium degenerative processes [17].

The expression of all three isoforms of TGF-β in the endometrium has been demonstrated in women. The highest level of TGF-β2 was observed in uterine stromal cells, whereas TGF-β1 and TGF-β3 were found to be present in both stromal and epithelial cells. The TGF-β1 was mainly observed in endometrial glands and in uterine fluid [18]. Changes in TGF-β expression and the signal transduction mechanism of this protein family may also lead to cell apoptosis. Chatzaki et al. [19] noted that TGF-β1 plays an important role in human endometrial stromal cells, where it can induce apoptosis via the FasL/Fas system. Caron et al. [10] showed that TGF-β1 is involved in the regulation of apoptosis in decidual cells by the inhibition of PI3-K/Akt pathways. TGF-β2 and TGF-β3 were also involved in the reduction in Akt phosphorylation, which might be an important mechanism that induces apoptosis. Eritja et al. [20] suggested that TGF-β/Smad signaling pathways play a crucial role in the regulation of cell proliferation or apoptosis of endometrial cells, and their alterations may lead to the development of cancer.

Cyclic changes in the level of expression were not observed for TGF-β1 and TGF-β2 cytokines, unlike TGF-β3, which is synthesized by the endometrial glands in the late secretory phase of the cycle [18]. Shooner et al. [21] showed that TGF-β1, TGF-β2 and TGF-β3 are expressed in the rat endometrium during decidual basalis regression and play an important role in the remodeling of the endometrium through the estrous cycle. Studies by Bukowska et al. [22] confirmed the expression of TGF-β isoforms in bitches’ endometrium during the estrous cycle. It was found that the expression of the analyzed transcripts depends on the phase of the estrous cycle. The highest level of TGF-β1, -β2 and -β3 expression was observed in the estrus phase, with lower expression in the proestrus, diestrus and anestrus phases.

Knowledge of changes in TGF-β expression in the canine endometrium is incomplete, especially in uterine diseases. Recently, transcriptome research has been conducted in an attempt to find functional and reliable diagnostic and prognostic markers that would indicate the development of CEH or pyometra at an early stage [23]. The aim of this study is to determine changes in the expression of TGF-β1, -β2 and -β3 in the endometrium of bitches with confirmed cystic growth of the endometrium glands and pyometra versus healthy tissue.

## 2. Materials and Methods

### 2.1. Uteri Sample Collections and Classification of the Study Groups

#### 2.1.1. Animals

Uteri samples for the study were obtained from 60 female dogs aged 1–10 years during routine ovariohysterectomy made at the request of their owners. All animals underwent precise clinical assessment. The bitches from which the material was collected had not previously received hormone therapy. More details about their status are presented in Table 1. The tissue samples were collected in line with standard veterinary protocols, with the consent of the owners. Following ovariohysterectomy, uteri with both ovaries were placed into 0.9% NaCl solution and transported at room temperature to the laboratory. Macroscopic observation, after longitudinal incision of the uterine horns, of factors such as the degree of redness, thickening of the endometrium, cysts on its surface, swelling or the presence and color of discharge allowed us to classify the uteri into study groups.

These classifications were then verified by histological analysis of slices. The control group (C; *n* = 20) consisted of uteri with a macroscopic lack of degenerative characteristics. Group I (GI; *n* = 20) consisted of uteri with CEH, where numerous multifocal serous-filled cysts were found on the surface of the endometrium. The second study group (GII; *n* = 20) was composed of significantly enlarged uteri, presenting a closed type of pyometra with cysts on the endometrium. The stages of the female dog estrous cycle were determined by vaginal smear. The control group, group I and group II consisted of females during diestrus.

Additionally, some observations were made on the collected ovaries. In the control group, GI and GII, normal ovaries with mature corpus luteum were observed during the diestrus phase. Pathological structures, mostly ovarian follicular cysts or, in several cases, cystic Corpora Lutea, were found in 4 bitches with CEH and 7 with CEH–pyometra.

The uteri distribution in the experimental groups was based on macroscopic evaluation and then confirmed by histological findings according to the classification suggested by De Bosschere [24].

#### 2.1.2. Clinical Evaluation of Animals

All animals underwent precise clinical assessment before ovariohysterectomy intervention. The uteri of bitches, which constituted the control group, came from animals that were completely healthy in the clinical examination and were in an optimum body condition. Only samples from bitches with no inflammatory alterations or cysts on the uterus were collected. In the control study groups, no significant abnormalities in the morphological or biochemical blood parameters were found.

In GI, most of the uteri used in the examination came from animals that were in an optimum body condition. Ultrasound examinations provided a description of the numerous multifocal cysts on the surface of the endometrium, filled with serous exudates, characteristic for CEH disorders. In bitches with mild to moderate CEH, no significant abnormalities in blood parameters were found. In cases where clinical symptoms were noted (GII), ultrasound examination confirmed pyometra with diverse intensities. During ultrasound examination, an enlarged uterus with convoluted, tubular horns containing anechoic or hypoechoic fluid and thickened endometrium were shown. The luminal contents were usually homogenous, and an accumulation of purulent secretions filled the organ. Furthermore, the presence of cystic structures with a thickened endometrium was noted as a symptom of CEH with pyometra.

Additionally, bitches diagnosed with pyometra presented other characteristic symptoms, with hematological changes specific to this disorder, as we have previously described [25].

### 2.2. Hematoxylin and Eosin Staining of Tissue Sections and Histological Examination

Contiguous sections were stained with H&E using procedures that we previously described [25]. Briefly, sections were deparaffinized in xylene (2 × 5 min) and rehydrated with successive washes in 100%, 96%, 80% and 70% ethanol. They were then stained with hematoxylin, rinsed with distilled water, rinsed with 0.1% hydrochloric acid in 50% ethanol, rinsed with tap water for 15 min, stained with eosin for 1 min and rinsed again with distilled water. The slides were then dehydrated with 95% and 100% ethanol, successively, followed by xylene (2 × 5 min) and mounted with coverslips. H&E sections were analyzed by light microscopy using an Olympus WX41 microscope (Hamburg, Germany). The Image Analysis Software “analySIS FIVE” (Version 1.0, Hamburg, Germany) was used to acquire images.

### 2.3. RNA Isolation and cDNA Synthesis

Samples of endometrium (100 mg) were homogenized with 1 mL of TRI–Reagent solution (Sigma, T9424 Darmstadt, Germany). Total RNA was extracted according to the manufacturer’s instructions. RNA integrity and quality were checked by 2% agarose gel electrophoresis. The amount and purity of RNA in the sample was measured using a Nano Drop 1000 spectrophotometer (Thermo Fisher Scientific Inc., Asheville, NC, USA). The RNA sample absorbance ratios at A260:280 nm were approximately 2.0. Each correct sample of total RNA (1 μg) was reverse transcribed to cDNA in a total of 20 µL using the Transcriptor First Stand cDNA Synthesis Kit (Roche Diagnostics, Boston, IN, USA).

The reverse transcription kit contained: SuperScriptTM II Reverse Transcriptase, First Strand Buffer, DTT, oligo-dT, dNTP mixture, RNase Inhibitor and nuclease-free water. The reaction was performed as described in the supplier’s protocol and involved the following steps: 65 °C for 10 min, 55 °C for 30 min, 85 °C for 5 min and was stopped at 4 °C. The obtained cDNA was stored at −20 °C until real-time PCR reaction.

### 2.4. Real-Time PCR Quantification

The levels of mRNA expression of TGF-β isoforms and reference genes were detected by real-time PCR (RQ-PCR) using the LightCycler 2.0 Real-Time PCR detection system (Roche Diagnostics, Mannheim, Germany). An RQ-PCR reaction was conducted using a TaqMan probe (Universal Probe Library, Roche Diagnostics, IN, USA) and the Light Cycler TaqMan Master System (04535286001, Roche Diagnostics, IN, USA). Relative gene expression was calculated by comparing the genes of interest with the reference gene GAPDH (glyceraldehyde 3-phosphate dehydrogenase) and beta-actin (ACTB) and was expressed in arbitrary units. In negative controls, samples of water were used instead of cDNA. Primers for the Universal Probe Library assay were designed using Probe Finder Software in the online Assay Design Center (Roche Diagnostic, IN, USA). The oligonucleotide sequence of primers (Sigma Aldrich, Darmstadt, Germany) and the probe sequence used for analysis are described in Table 2. The real-time PCR reaction mix (20 μL) contained: 4 μL LightCycler Master Mix 5×, 1 µL primers (200 nM each: forward and reverse), 1 μL suitable TaqMan probe (200 nM) 9 µL water, and 5 µL template (equivalent to 12.5 ng RNA). Amplification was preceded by an initial enzyme activation step for 10 min at 95 °C. The PCR reaction parameters were as follows: 40–45 cycles of 10 s at 95 °C, 30 s at 60 °C and 1 s at 72 °C. Cooling was carried out for 30 s in 40 °C. Blank reactions without a DNA template did not show any fluorescence signal. For quantification, standard curves consisting of serial dilutions of the appropriate cDNA were drafted to evaluate amplification efficiency. Each sample was amplified in triplicates. The obtained data were calculated using ΔΔCt methods [26].

### 2.5. Western Blotting

Samples of endometrium were homogenized in RIPA buffer (50 mmol/L Tris–HCl, pH 8.0 with 150 mmol NaCl, 1.0% NP-40, 0.5% sodium deoxycholate, 0.1% SDS, 10 mmol/L NaF and 1 mmol/L Na3(VO4)), containing protease and phosphatase inhibitor cocktails (Roche Diagnostics, IN, USA). The lysates were centrifuged (14,000× *g*, 10 min, at 4 °C), and the supernatants were collected and stored at −80 °C. The protein concentration was measured using a BCA Protein Assay Kit (Thermo Scientific, Rockford, IL, USA). Proteins (30 μg per lane) were diluted in Laemmli Sample Buffer (BioRad, Munich, Germany) and denatured at 95 °C for 5 min. The samples were separated on 15% Tris–HCl SDS-PAGE gel and blotted onto a PVDF Transfer Membrane (Thermo Scientific, Rockford, IL, USA). The membranes were blocked in 5% BSA in TBST (50 mmol/L Tris, 100 mmol/L NaCl, 0.1% Tween 20, pH 7.4) overnight at 4 °C. Then, membranes were incubated (overnight) with anti-TGFβ1 (1:500 dilution, sc-31609, Santa Cruz Biotechnology, Dallas, TX, USA), anti-TGFβ2 (1:250 dilution, sc-90, Santa Cruz Biotechnology, Dallas, TA, USA) and anti-TGFβ3 (1:250 dilution, sc-166833, Santa Cruz Biotechnology, Dallas, TA, USA) in TBST supplemented with 1% BSA at 4 °C. Membranes were then washed with TBST and incubated with the secondary anti-mouse IgG HRP-linked goat antibody (1:5000 dilution, sc-2005, Santa Cruz Biotechnology, Dallas, TX, USA) in TBST for 1 h at room temperature. GAPDH (1:1000 dilution, sc-47724, Santa Cruz Biotechnology, Dallas, TX, USA) expression was used as a loading control. Proteins were visualized by incubating the membranes in Immobilon Forte Western HRP substrate (WBLUF0100, Merck, Darmstadt, Germany).

The signals were captured by the VersaDoc Imaging System (Bio-Rad Laboratories, Munich, Germany), and then the intensity was quantified using Quantity One 1-D Analysis Software (Bio-Rad Laboratories, Munich, Germany).

### 2.6. Statistical Analysis

Statistical analysis was performed using GraphPad Prism (GraphPad PRISM, Version 5.0, San Diego, CA, USA). The Shapiro–Wilk test was performed for testing the normality of data. The Kruskal–Wallis test for comparisons of significance among all groups (C, GI, GII), and then pairwise comparisons of groups, using a Wilcoxon test, were performed. All data were represented as means ± SEM. The critical value for significance in all the experiments was *p* < 0.05.

## 3. Results

### 3.1. Histopathological Analysis of Uteri: Normal and with Morphological Abnormalities

Control group—uteri without abnormalities.

**Figure 1:**
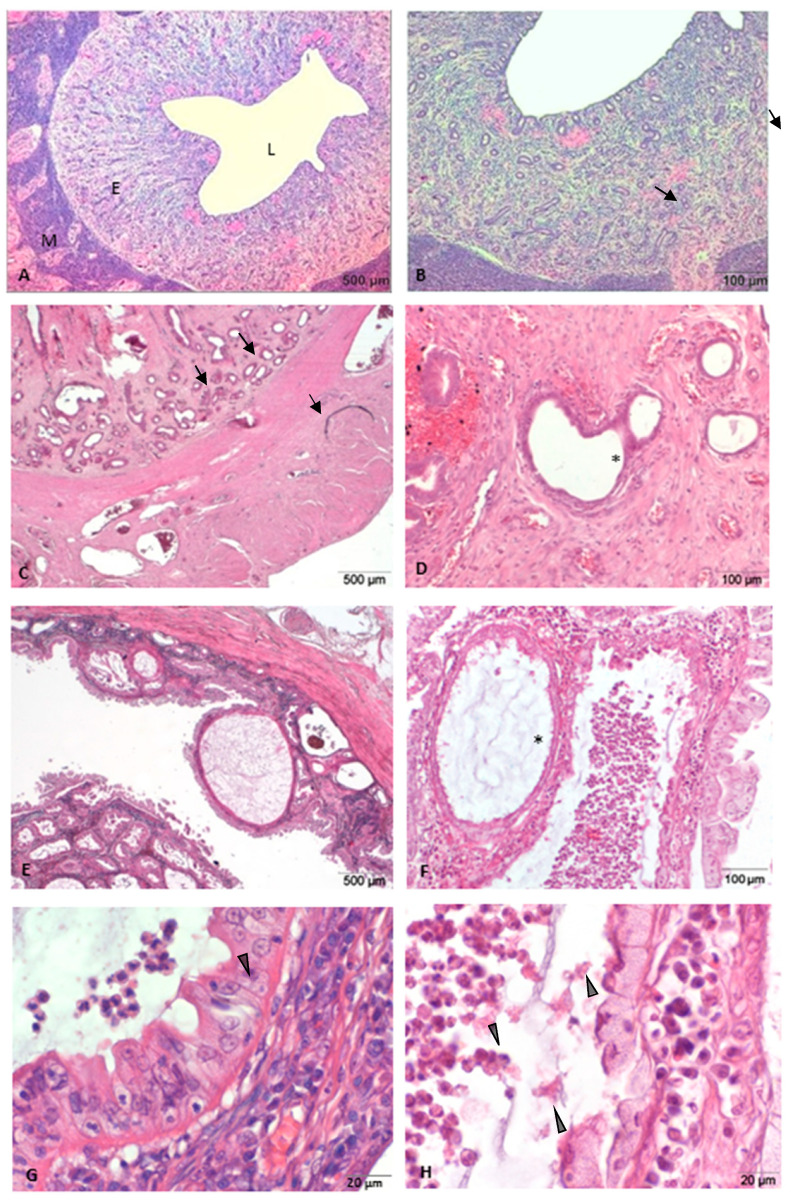
Representative photomicrographs of canine endometrial tissue with various proliferative, degenerative and inflammatory changes. (**A**,**B**) Physiological endometrium (control group); (**C**,**D**) cystic endometrial hyperplasia; (**E**–**H**) CEH –pyometra complex. L, uterine lumen; E, endometrium; M, myometrium; black arrows, endometrial glands; asterisk, endometrial cysts; arrowheads, neutrophils infiltrations. H&E magnification: (**A**,**C**,**E**) × 40; (**B**,**D**,**F**) × 100; (**G**,**H**) × 400.

The assessment of microscopic slices confirmed the absence of morphological abnormalities of the endometrium and myometrium, in accordance with preliminary macroscopic evaluation. The morphological image was typical of the diestrus stage in the reproductive cycle of bitches (Figure 1A,B).

Group I—uteri with cystic endometrial hyperplasia (CEH).

Microscopically, a mild to moderate swelling of the endometrium was observed. Endometrial glands of different sizes were focally or multifocally arranged in the endometrium. Atrophic glandular epithelium were revealed. Additionally, in several cases, hemorrhage of the endometrium was observed (Figure 1C,D).

Group II—uteri with CEH- pyometra complex.

Histologically, multifocal cystic endometrial hyperplasia, lymphocytes and neutrophil migration into the lumen of endometrial glands was detected. In all uteri, there was extensive purulent inflammation of the endometrium, which was usually diffuse or multifocal. Moreover, in several cases, we identified interstitial fibrosis of the endometrium, adenomyosis and endometrial hemorrhage in the tissue section (Figure 1E,F).

### 3.2. mRNA Expression Profile of TGF-βs in the Canine Endometrium

**Figure 2:**
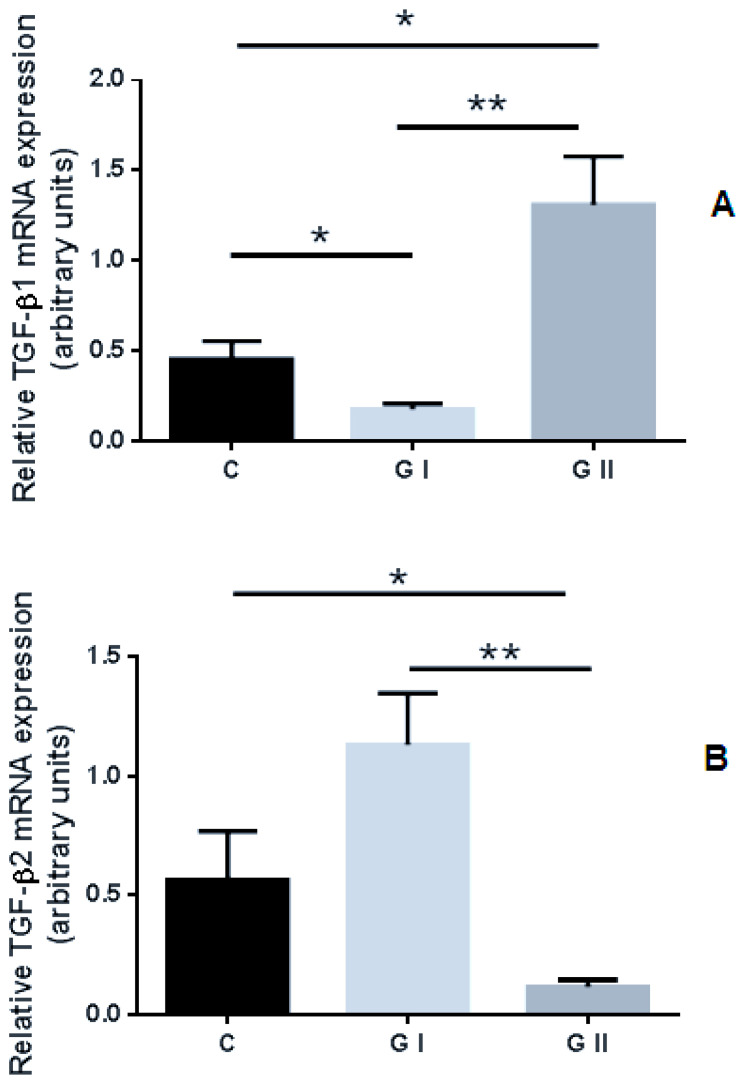

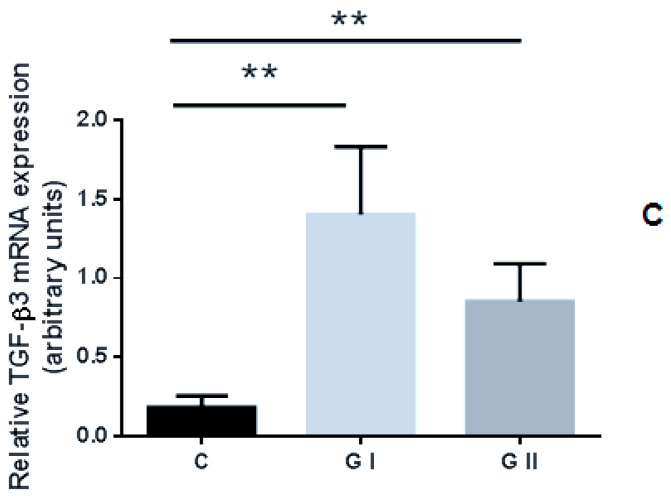
Relative expression of (**A**) TGF-β1, (**B**) TGF-β2, and (**C**) TGF-β3 in canine endometrium with various proliferative and degenerative changes: cystic endometrial hyperplasia (GI) and CEH–pyometra (GII) compared to the standard physiological endometrium (C, control group). The data were normalized by dividing the expression of target genes by the expression of the housekeeping gene (GAPDH). All treatments were performed in triplicates and bars represent the mean ± SEM. The asterisks indicate significant differences as follows: *, *p* < 0.05; **, *p* < 0.01.

Statistically significant differences in the expression level of TGF-β1, TGF-β2 and TGF-β3 genes among endometrium obtained from the control and studied groups were noted. Increased expression of TGF-β1 mRNA was recorded in the endometrium of bitches with CEH–pyometra (Group II; GII) compared to the control group of clinically healthy bitches (*p* < 0.01) and group I, suffering from cystic endometrial hyperplasia (*p* < 0.05). A lower expression of TGF-β1 mRNA in the endometrium collected from CEH (Group I; GI) compared to the control group (*p* < 0.05) and GII (*p* < 0.01) was observed (Figure 2A). The mRNA expression of TGF-β2 was reduced in the group of bitches in group II compared to the control group (*p* < 0.05) or GI (*p* < 0.001), (Figure 2B). Increased levels of TGF-β3 mRNA expression were noted in both the studied groups compared to the control group (*p* < 0.05; *p* < 0.001). There were no significant differences (*p* > 0.05) in the expression of TGF-β3 mRNA between the analyzed GI and GII groups (Figure 2C).

### 3.3. The Protein Production of TGF-β Isoforms in the Canine Endometrium

**Figure 3:**
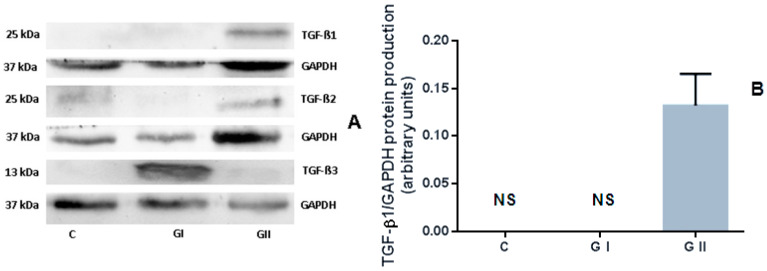

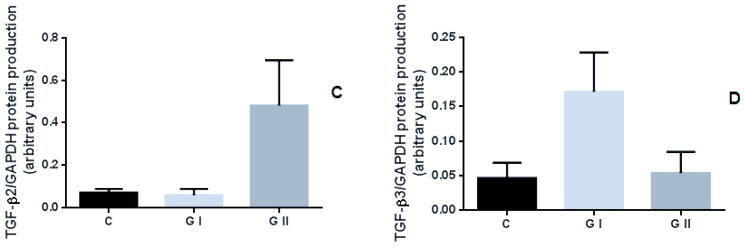
TGF-β1 (**B**), TGF-β2 (**C**), and TGF-β3 (**D**) protein production in bitches’ endometrium samples with cystic endometrial hyperplasia (GI) and pyometra (GII) compared to the physiological endometrium (**C**, control group). (**A**) Representative blots for TGF-β and GAPDH. The data were normalized to glyceraldehyde-3-phosphate dehydrogenase (GAPDH). The bars represent the mean ± SEM, in arbitrary units. The TGF-β1 (**B**) and TGF-β2 (**C**) signals were identified as mature dimers (25 kDA); for the TGF-β3 (**D**) signal, mature monomer (13 kDA) was detected. NS, no protein signal.

The results showed that TGF-β1 protein production occurred only in the endometrium of CEH–pyometra bitches. No signal was visible in the control group or GI (Figure 3A). There were no statistically significant differences (*p* > 0.05) in the TGF-β2 and TGF-β3 protein production levels among the control and the other studied groups or between GI and GII (Figure 3B,C). However, the results indicated a higher protein production of TGF-β2 in bitches from GII compared to GI or the control group. The highest TGF-β3 protein level was found in bitches suffering from CEH (GI).

## 4. Discussion

The role of TGF-β signaling in uterine epithelial cells has not yet been fully defined in mammals. Due to the significant participation of the discussed factors in cellular processes related to proliferation or differentiation, TGF-β factors have been identified as potential modulators of many changes occurring in the endometrium during the estrous cycle and pregnancy [10,27].

There are no data in the literature describing the expression of TGF-β in the canine endometrium with pathological disorders. In our study, significant differences in the expression levels of TGF-β1, TGF-β2 and TGF-β3 in bitches with a CEH or CEH–pyometra endometrium versus healthy uterus were noted. The obtained results indicate that the uteri of bitches affected by CEH–pyometra complex had a significantly higher level of TGF-β1 expression (mRNA and protein expression) compared to the control group and bitches with CEH. Pathological uteri with CEH–pyometra included tissues characterized by an advanced endometrial inflammatory process [25,28]. In presented histopathological studies, in bitches affected by CEH–pyometra, a significant degree of fibrosis of the endometrium was also observed in individual cases, which could confirm the involvement of TGF-β1 in both inflammatory and degenerative processes. The uterus with CEH was characterized by a lower level of TGF-β1 mRNA expression than in the other groups. However, no protein signal was detected in the endometrium collected from these bitches. Therefore, the TGF-β1 cytokine seems to have a much lower implication in the development of CEH (represented mostly proliferative changes in a uterus) compared to the pyometra disease in canines.

Various chronic inflammatory diseases are characterized by inappropriate or dysregulated activity of TGF-β [29]. TGF-β1 is the main regulator of immune protection and immune pathology. The role of endometrial TGF-β1, IL-10 and IL-17 in chronic endometritis (CE) was investigated by Wang et al. [30]. They analyzed a possible relationship between autophagy and local cytokines in women with CE and repeated implantation failure (RIF). In this study, women with CE had significantly decreased TGF-β1 and IL-10 expression in the endometrium, with additionally increased IL-17 expression levels. CE led to the functional deficiency of Treg cells, accompanied by increased autophagy. Consequently, inflammation and fibrosis decreased endometrial sensitivity and the pregnancy rate. The results suggested that regulation of autophagy could be the key to regulating local immune responses and improving the implantation rate in patients with endometritis.

The potential role of TGF-β signaling in uncontrolled epithelial cell proliferation and endometrial hyperplasia in mouse model was investigated by Gao et al. [13]. It was found that ovariectomized Tgfbr1 (TGF-β receptor 1) conditional knockout mice are characterized by increased uterine epithelial cell proliferation and display increased expression of fibroblast growth factor 10 (FGF10). Moreover, the treatment of primary uterine stromal cells with TGF-β1 significantly reduced FGF10 mRNA expression in the uterus [13]. The authors speculated, that TGF-β signaling pathways may contribute to regulating/stimulation of angiogenic gene expression, leading to the development of uterine gland disorders or endometrial carcinoma [13,31].

Additionally, numerus studies have confirmed the prominent role of TGF-β1 in the immune response and regulation of endometriosis pathology [32,33,34,35,36]. The concentration of TGF-β1 in the peritoneal fluid (PF) and serum was higher in women with endometriosis compared with healthy individuals. In case of TGF-β2 and TGF-β3, the results are inconsistent. However, TGF-β2 and TGF-β3 could also play an important role in immune regulation, which needs further investigation [36]. The TGF-β1 is considered one of the most important mediators of fibrogenesis [37]. It is assumed that TGF-β1 inhibits NK cell activity and induces angiogenesis and proliferation of endometrial stem cells [38].

The increased expression of TGF-β2 or TGF-β3 in endometrial tissue is considered the basis of the pathophysiology of endometrial diseases in women. In the molecular mechanism of adenomyosis-related fibrogenesis, evidence suggests that the etiology of adenomyosis is linked to TGF-β [39,40]. Higher levels of TGF-β2 were noted in endometrial epithelial cells of adenomyosis lesions than in control women’s samples. Yoo et al. suggest that TGF-β2 plays an important role in adenomyosis development as a direct target of β-catenin [41].

The available literature shows that high concentrations or overexpression of TGF-β mediators may be responsible for the pathophysiology of uterine fibroids (UFs) [42]. TGF-β3 reduces human endometrium receptivity by decreasing the expression of BMP-2 receptors and has a significant role in overproduction of the ECM (extracellular matrix) [43]. The TGF-β3 level was higher in human leiomyoma cells. This cytokine promotes leiomyoma development by stimulating cell growth and fibrogenic process [44].

In the present study, the opposite situation (compared to TGF-β1 expression) was observed for TGF- β2 mRNA. The uterus with CEH–pyometra was characterized by a lower level of expression of this cytokine than in the other groups. These results may suggest different mechanisms of TGF-β2 regulation in tissues with an advanced inflammatory process. Interestingly, the expression profile of the third isoform, TGF-β3, was significantly higher in all pathological groups compared to the endometrium collected from the healthy uteri. There were no differences in the expression levels of TGF-β3 mRNA between the CEH and CEH–pyometra bitches. Increased expression levels of TGF-β1 or TGF-β3, in the case of bitches with CEH or pyometra, compared to healthy females, may indicate the similar involvement of these cytokines in the development of degenerative changes in the uterus. However, further investigation is warranted to study this hypothesis.

The changes in TGF-β2 and TGF-β3 mRNA levels were not confirmed in protein detection. However, for both cytokines, the results were not statistically significant. The highest TGF-β2 protein levels were shown in the pyometra group compared to CEH or healthy animals. For TGF-β3, the maximum protein levels were detected in bitches suffering from CEH. A lack of correlation in TGF-β mRNA with protein expression has previously been reported. The levels of TGF-β1 mRNA did not correlate with the distribution of TGF-β1 protein, measured by immunohistochemical staining [45]. These findings emphasize the importance of evaluating TGF-β at the protein level. The biological activity of the three isoforms of TGF-β was similar in most in vitro experiments, while collected evidence suggests differences in their in vivo competencies [46].

As we have recently described, a typical CEH–pyometra uterus presented with a major degree of endometritis, mainly purulent, and chronic, inflammatory processes in the endometrial stroma with the infiltration of inflammatory cells [25]. Additionally, in several cases, multifocal fibrosis of the endometrium and adenomyosis was observed. The variability of gene expression of all TGF-β isoforms presented in this study and their correlation with specific pathological changes in the canine uterus require further examination. The presented results have some limitations; to confirm the role of TGF-β proteins in CEH–pyometra diseases, it would be worth extending the analysis to investigate the cytokine serum level, localization assay or differences in individuals’ ages and breed type.

## 5. Conclusions

The results of this study expand the knowledge of molecular factors involved in the development of the pathogenesis of “CEH–pyometra complex” in bitches. Among all TGF-β isoforms, TGF-β1 seems to be a potential marker helpful in the identification of canine uterine disorders. Further analysis is required to determine the involvement of TGF-β2 or TGF-β3 in the processes of repair, inflammation and excessive cell proliferation in canines suffering from CEH–pyometra complex.

## Figures and Tables

**Table 1:** Details of the animals (control group, cystic endometrial hyperplasia (CEH) and CEH-pyometra) used in the research.

Characteristics	Control Group	Group I CEH	Group II CEH–Pyometra
Number of bitches	*n* = 20	*n* = 20	*n* = 20
Age (years)			
max	3	10	10
min	1	3	2
mean ± SD	1.92 ± 0.97	6.4 ± 2.4	7.9 ± 2.2
Body weight (kg)			
max	40	42	46
min	2	3	5
mean ± SD	19.8 ± 11.1	20.4 ± 9.8	18.4 ± 14.1
Breed			
crossbreed	12	8	11
in breed type	8	12	9
			

**Table 2:** Oligonucleotide sequences and probe number use for real-time PCR.

Gene Name	Primer Sequences 5′–3′	Gene Accession No.	Catalog No.	Product Size (bp)
TGF-β1	F: ATGAGCCCAAGGGTTACCA R: GTCCAGGCTCCAAATGTAGG	NM_001003309	04688546001	65
TGF-β2	F: GCAGCAAGACGATAATCACG R: TCTTGTCGCTGTCGTCCTC	XM_545713.3	04688678001	67
TGF-β3	F: CTGGCCCTTTACAACAGCAC R: CGACTCGGTGTTTTCCTGAG	DQ310186.1	04686993001	87
GAPDH	F:GCTGGGGCTCACTTGAAA R:GTTCACGCCCATCACAAAC	NM_001003142.1	04688678001	87
ACTB	F:CTGGACTTCGAGCAGGAGA R:CCGTCGGGTAGTTCGTAGC	AF021873.2	04689038001	73

## Data Availability

All data sets obtained and analyzed during the experiment are available up on reasonable request from the respective author.

## Abbreviations

BMP-2	Bone morphogenetic protein 2
CE	Chronic endometritis
CEH	Cystic endometrial hyperplasia
CL	Corpus luteum
ECM	Extracellular matrix
FGF10	Fibroblast growth factor 10
GAPDH	Glyceraldehyde-3-phosphate dehydrogenase
Gf	Growth factors
IL	Interleukin
NK	Natural killer
P4	Progesterone
PF	Peritoneal fluid
PGF2α	Prostaglandin F2α
PI3-K/Akt	Phosphoinositide-3-kinase/Protein kinase B
RIF	Repeated implantation failure
TGF-β	Transforming growth factor beta
Tgfbr1	Transforming growth factor beta receptor1
UFs	Uterine fibroids
